# Development and Validation of a Scale for Interaction between Parents and Coaches of Middle and High School Golf Players

**DOI:** 10.3390/ijerph18179419

**Published:** 2021-09-06

**Authors:** Jae-Wook Hwang

**Affiliations:** Department of Physical Education, Yonsei University, Seoul 03722, Korea; winnika1@naver.com

**Keywords:** parent, coaches, interaction, development, validation

## Abstract

The purpose of this study was to develop a measurement tool for the interaction between parents and coaches of middle and high school golfers, and to verify its validity. A total of 563 parents participated in the study. Based on the results of preliminary item production, item analysis, reliability analysis, exploratory factor analysis, identification factor analysis, job uniformity analysis, and potential mean comparison analysis, the initial 70 items were constructed according to the conceptualization of parent–coach interactions. The first and second item reduction processes and preliminary surveys were conducted through expert meetings to produce the final 40 items of parent–coach interactions. After eliminating 20 items through question analysis, reliability analysis, and exploratory factor analysis, the final three factors of parent–coach interaction were extracted, and the conformity index for the middle and high school golfers’ parent-to-coach interaction tool was checked. Then, to ensure the external validity of the developed parent–coach interaction measurement tool, a construct equivalence analysis was conducted to demonstrate that the tool can be applied equally to parents and coaches. In summary, the tools for measuring the interaction between the middle and high school golfers’ parents and coaches were developed in 20 questions, three factors for communication, qualification, and support, and it was confirmed that the developed measuring tools could all be applied equally through a latent mean analysis. Parents and coaches are key variables that can affect a player’s performance; thus parents and coaches’ interaction measuring tools can be provide positive information not only for golfers but also for players of various other sports.

## 1. Introduction

Middle and high school student-athletes experience various problems in the process of becoming adult athletes, and to overcome these difficulties, they essentially require positive interactions with their parents and coaches [1]. In the field of sports, parents and coaches play an important role in the process of grooming athletes, as they discover, guide, and support athletes’ talents from an early stage [2,3]. They play a pivotal role in improving athletes’ performance by creating a foundation for them to focus on sports and establishing a multi-faceted support system [4,5]. From this point of view, while the individual role of parents and coaches is important for athletes’ performance enhancement, it is important to recognize that the support provided by the desirable interaction of these two groups is also essential.

Parents and coaches are closely involved in student athletes’ lives in complex and diverse areas, ranging from overall daily life to improvement of performance [6]. In particular, the significance of parents’ influence in a particular sport, say golf, is easily recognizable through various sources in mass media [7]. Parents of golf players perform the role of parent as well as the helper in many ways, including acting as managers, caddies, coaches, and counselors, to the extent that the term “golf daddy” has been newly coined in Korea to denote the significance of their contribution in the players’ life [8].

Due to the close nature of the relationship between parents and athletes, parents’ interests and expectations affect athletes’ performance, which is their top priority [9]. The reason that Korean golfers were able to succeed on the ladies professional golf association (LPGA) tour was due to their parents’ passion and various experiences gathered from studying abroad; the foreign media at home and abroad concluded that the reason for their excellent performance was their parents’ dedication [8]. However, excessive interference and expectations of parents may also create psychological pressure on athletes, and parents’ reprimands and verbal abuse can negatively affect their performance.

Parents of middle and high school golfers help their children grow throughout their school life and seek efficient ways to improve their performance. Therefore, the influence of parents on athletes’ performance cannot be overlooked. However, the role of coaches is also an indispensable part of athletes’ lives and performance [10]. The interactions between coaches and athletes play an important role in improving athletes’ psychological and social development and performance [11]. As shown in Jowett’s model [12], coaches’ and athletes’ individual differences, roles in sociocultural contexts, and interpersonal characteristics can affect athletes’ performance.

With increasing interest in interpersonal interaction in the field of sports, many studies on the interaction between athletes, parents, and coaches have been conducted; these studies primarily targeted the athletes. Prior studies on athlete-parent interactions were aimed at assisting the athletes and identifying positive changes through the overall parental support system [13,14]. Hwang et al. [15] also identified the general aspects of support for athletes and parents’ common influence on middle and high school golfers, as well as parents’ interactions.

Prior studies show that athletes’ relationship with parents and coaches is not a one-way, but a two-way process [15,16,17]; however, there is a lack of research on the relationship between parents and coaches, particularly, how the interaction between parents and coaches can affect athletes. While parents and coaches pursue a common purpose, that of improving athletes’ performance, there are clear differences in the tasks, roles, and behaviors performed by them during competitions, training, and daily life; moreover, parents’ and coaches’ thoughts and perspectives can be subjective. The relationship and interaction between the two also change easily depending on the situation. Discord between parents and coaches can cause unintended problems and negatively affect athletes’ career choices, school attendance, study, and athletic performance [18]. Therefore, it should be noted that desirable interactions between parents and coaches can help athletes improve their performance by proactively preventing any disturbances and assisting in the efficient execution of training plans.

A reasonable inference that a positive relationship between parents and coaches could affect a player’s performance can be derived from qualitative research on interactions between parents and coaches of middle and high school student golfers [17]. In this study, we conducted open-ended questions and in-depth interviews with each group (parents and coaches) to identify the conceptual structure of parent–coach interactions, and explored common factors of their interactions based on the responses from each group.

With increasing academic interest in interpersonal interaction in sports, interaction measurement tools have been developed intermittently to overcome the challenge of quantitatively measuring these interactions [19]. Foreign studies on the relationships between athletes, parents, and coaches have some limitations in that they view the changes in dependent variables based on their interests rather than understanding the interactions; whereas, domestic interaction studies have focused on improving athletes’ performance from the perspective of players, parents, and coaches.

Stephan [20] argued that the main factors that determine the quality of athletes’ performance are the interactions between players, parents, and coaches. Thus, so far, the study of interactions in the sports domain has been mainly from the perspective of athletes, parents, and coaches, and the study of the interaction between parents and coaches is relatively in the early stages. Based on the theoretical evidence [21] that desirable interactions between parents and coaches can have a positive impact on players, a measurement tool is needed to provide a more meaningful and concise understanding of these interactions. In order to develop measurement tools, we can set up research questions about the question composition and factor structure of parent–coach interactions and the external validity of developed interaction measurement tools based on the previous description.

The purpose of this study was to reconstruct the contents of general interactions between parents and coaches, based on the study of our group [17], and to develop an interaction measurement tool and ensuring its feasibility. Furthermore, the development of the parent–coach interaction measurement tool can provide information that can positively improve the relationship between parents and coaches, ultimately contributing to the performance of student-athletes, a common goal of the two groups. The research hypotheses for identifying the purpose of this study are as follows. First, content analysis will allow us to explore the question composition and the factor structure of parent–coach interactions. Second, the external validity of the developed interaction measurement tools can be obtained.

## 2. Materials and Methods

### 2.1. Study Participants

The parents of middle and high school children were included in the study, while golf coaches comprised professional golf youth directors and coaches, individual golf lessons coaches, and coaches from golf practice centers and golf academies. A preliminary survey was conducted on 40 study participants (20 parents and golf coaches). A total of 150 parents and 150 golf coaches were recruited to develop the interaction measurement tool, excluding 16 participants whose data were not suitable for analysis. To validate the tool’s feasibility, data were collected from a total of 250 participants (125 parents and 125 golf coaches), and the process was carried out to secure external validity, excluding 11 cases with inappropriate data. The selection of subjects for the pilot test is reviewed with approximately one-tenth of the number of subjects selected for research in this study, and it was set as the standard for precedent research on the development of measurement tools [16]. The basis for the study’s development and validation of the test paper was selected in proportion to the sample size of the population. For factor analysis, the size of the required collection is based on the range of stability [14,17] if the number of cases is approximately 200 or more, or the ratio of the number of cases and measurement variables is more than 5:1.

### 2.2. Ethical Considerations 

The author has completed a research ethics course conducted by the Research Ethics Review Committee of Yon Sei University to ensure the ethical aspects of participation. Participants were notified of the background, purpose, and methods of the study, the method, side effects or risks involved in study participation, use of personal information, and confidentiality (IRB no. 7001988-202009-HR-655-03).

### 2.3. Development Method and Procedure

The following procedures were carried out to develop an interaction measurement tool to measure the interaction between parents and coaches of middle and high school golfers. To collect parental data, the researcher visited parents’ residences or workplaces in Seoul, Incheon, and Gyeonggi-do. To collect data from coaches, the researcher visited golf training centers and golf academies in Seoul, Incheon, Gyeonggi-do, Gangwon-do, and Chungcheongnam-do.

**Figure 1:**
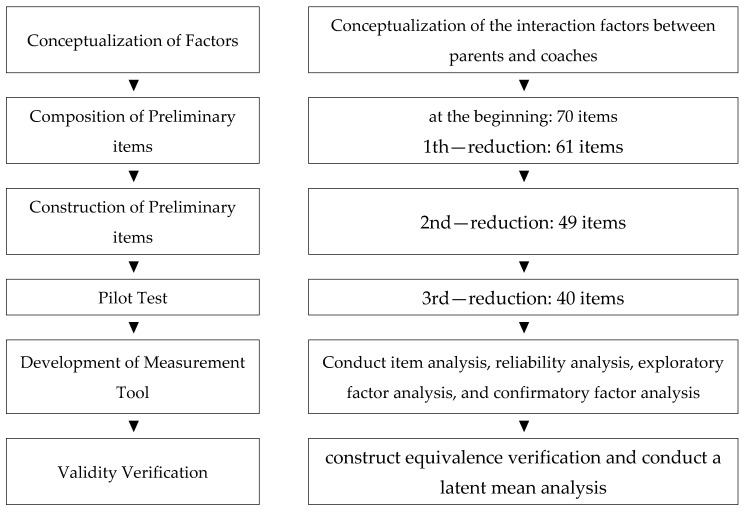
Procedure for developing a parent–coach interaction measurement tool.

Based on the results of our group [17], the first and second rounds of item reduction were conducted in a meeting with several experts (a sports psychology professor, two sports psychologists, a parent, a golf coach, and the researcher) to verify the appropriateness and validity of the preliminary questionnaire. In the preliminary survey, 40 parents and coaches were recruited; 284 parents and coaches were surveyed to conduct question analysis, reliability analysis, exploratory factor analysis, and confirmatory factor analysis to develop a measurement tool for parent–coach interaction. In addition, 239 parents and coaches were assessed for job/role equality to determine whether the tool could be applied equally to each group, and a potential average comparison analysis was conducted to determine whether there were any differences between each factor. Figure 1 shows the procedure for this study.

### 2.4. Data Processing and Statistical Analysis

The data collected in this survey were calculated using SPSS and AMOS (Version 27.0, SPSS, Inc., Chicago, IL, USA) programs to calculate the means, standard deviations, kurtosis, and skewness, and to conduct reliability analysis and exploratory factor analysis. Models 1, 2, and 3 were derived through a confirmatory factor analysis to verify the goodness-of-fit index of the interaction measurement tool. To verify the external validity of the developed tool, the AMOS program was used to conduct a construct equivalence verification and latent mean analysis.

## 3. Results

### 3.1. Development of an Interaction Measurement Tool for Parents and Coaches of Middle and High School Golfers

#### 3.1.1. Composition of Preliminary Questions

The preliminary items were produced based on the results of prior research by our group [17]. The initial questionnaire comprised 70 items, designed to be easily understood by parents and coaches, and included most of the terms commonly used in golf. Through the first and second rounds of the reduction process, the 70 items were reduced to 49, and a pilot test was conducted with 40 parents and coaches to derive the final 40 items.

The 70 questions generated through preliminary study on interaction and expert discussion were reviewed repeatedly by one professor of sports psychology, two Ph. Ds in sports psychology with experience in developing measuring tools. To verify the terms used on the golf field, and the simplicity, meaning, and expressiveness of the items, the experts’ meeting included one player, one parent, one coach, and one Korean teacher to reduce the number of questions.

To check the consistency of the responses, the three items (No. 38, 39, and 40) were selected as reverse scoring items.

#### 3.1.2. Item Analysis

In the development of measurement tools, researchers appropriate that items with a mean of ≥4.5 on a five-point scale or a standard deviation of 0.1 or higher, and items with a response rate of 50% or more on a scale should be deleted [15]. The average of the 40 items of parent–coach interaction was 2.51–3.58, with no items above 4.5. In addition, the standard deviations ranged from 0.83 to 1.15, with no items below 0.1; therefore, no items were deleted according to the mean and standard deviation criteria. There were no more than ±1.0 items for kurtosis and skewness, but a total of three items were eliminated in accordance with the criteria for response rate: 25 (56.7%), 29 (68.7%), and 39 (62.3%), with a 50% response rate (Table 1).

#### 3.1.3. Reliability Analysis

A reliability analysis (Cronbach’s α) was conducted with 37 items, excluding three items that were found to be inappropriate for the reference value through item analysis. As a result of the first reliability analysis, eight items (No. 3, 7, 12, 18, 19, 20, 36, and 38), whose deletion led to an increase in the α coefficient, were deleted; three more items were deleted through the second reliability analysis (No. 15, 10, and 23). A repeated tertiary reliability analysis was performed, where one item (No. 21) which resulted in an increase in the α coefficient was deleted, and no item with a correlation of 0.30 or less between the items. Through reliability analysis, 12 of the 37 items were deleted, and the overall reliability coefficient was found to be 0.909.

#### 3.1.4. Exploratory Factor Analysis

Exploratory factor analysis was conducted with a total of 25 items, except for 15 that did not meet the criteria in the item analysis and reliability analysis. The exploratory factor analysis estimates the factors with maximum likelihood analysis, and the rotation method uses direct oblimin, which presupposes the correlation of the factors. Bartlett’s spherical verification results (χ^2^ = 1684.223, df = 190, *p* = 0.000) and Kaiser–Meyer–Olkin were 0.903.

In the exploratory factor analysis, factors with eigenvalues greater than one were extracted; items with factor loadings less than or equal to 0.40 and with factor loadings greater than two, were deleted. After several repeated exploratory factor analyses, 20 items for three factors (communication, talent, and support) were extracted through the final exploratory factor analysis after deleting a total of five items for the communication factor (No. 5 and 8) and support factor (No. 14, 24, and 30). The factor loadings of the three subfactors extracted from the final 20 items ranged from 0.412 to 0.773; the talent factor ranged from 0.526 to 0.623, the communication factor ranged from 0.412 to 0.733, and the support factor from 0.412 to 0.773. An analysis of the reliability of each subfactor showed a value of 0.787 for the communication factor, 0.751 for the talent factor, and 0.769 for the support factor (Table 2).

#### 3.1.5. Confirmatory Factor Analysis

We conducted a confirmatory factor analysis using AMOS 20 with 20 items of the three factors extracted from the exploratory factor analysis. Models 1, 2, and 3 were developed to verify the validity of the interaction measurement tool for parents and coaches of middle and high school golfers. Model 1 identified the fit of a single factor as an initial research model, and Model 2 identified the factor structure with 20 items of Factor 3. Model 3 was validated through hierarchical secondary models to determine whether the three subfactors were suitable for measuring the interaction between parents and coaches of middle and high school golfers.

Confirmatory factor analysis for Model 1 showed the model’s goodness-of-fit index as: Q = 2.278, TLI = 0.842, CFI = 0.859, and RMSEA = 0.067. The Q and RMSEA values were good, but the goodness-of-fit indices for the TLI and CFI values were not satisfactory. Thus, Model 1 for 20 items of the single factor was found to be a nonconforming model. Confirmatory factor analysis for Model 2 showed the model’s goodness-of-fit index as: Q = 1.776, TLI = 0.904, CFI = 0.916, and RMSEA = 0.052, which proved that Model 2 for 20 items of the three factors was a suitable model. Confirmatory factor analysis for Model 3 showed that the model’s goodness-of-fit index (Q = 1.776, TLI = 0.904, CFI = 0.916, and RMSEA = 0.052) was the same as the goodness-of-fit index of Model 2. Thus, Model 3, a hierarchical quadratic model, was accepted and the relationship between the three primary factors was found to be highly descriptive by one secondary factor (Table 3).

### 3.2. External Validity Verification of the Measurement Tool

#### 3.2.1. Construct Equivalence for Interaction between Parents and Coaches

Construct equivalence analysis was conducted to determine whether the developed interaction measurement tool could be applied equally to parents and coaches. Before the analysis, the structural suitability of each group (parents and coaches) was determined. According to the analysis, the coaches’ group (Q = 1.959, TLI = 0.915 CFI = 0.925, RMSEA = 0.089) showed a satisfactory goodness-of-fit index, but the parents’ group (Q = 1.927, TLI = 0.893, CFI = 0.906, RMSEA = 0.089) did not. However, Bagozzi and Dholakia point out that a model is evaluated as good, as per the goodness-of-fit criterion, if the TLI and CFI are within 0.80 to 0.90; this indicates that the study results can be considered acceptable [22]. As each group’s goodness-of-fit index was good, uniformity of form, measurement, intercept, and factor variance were performed to ensure that the interaction measurement tool could be applied equally to parents and coaches.

Configural invariance (Q = 1.942, TLI = 0.906, CFI = 0.917, RMSEA = 0.063) showed good results. Therefore, both parents and coaches were found to have a common model for the middle and high school golfers’ parent-to-coach interaction questionnaire. The goodness-of-fit index of measurement invariance (Q = 1.958, TLI = 0.904, CFI = 0.911, RMSEA = 0.064) was good, and the results of using the χ^2^ value to compare Model 1 and Model 2 (⊿χ^2^ [df = 17, N = 239] = 38.676, *p* < 0.001) showed a difference between the models, which was rejected. However, because TLI, CFI, and RMSEA are indices that consider the simplicity of the model, it can be said that the equality constraint is established if the index of the model with measurement invariance constraints is not worse than the shape uniformity model index [23]. Comparison of TLI, CFI, and RMSEA differences between Model 1 and Model 2 showed a slight difference (A), such that the measurement invariance of Model 2 was established, demonstrating that the parent–coach interaction questionnaire for middle and high school golfers was common for both groups. The goodness-of-fit index of scalar invariance was as follows: Q = 1.890, TLI = 0.911, CFI = 0.913, and RMSEA = 0.061. Using χ^2^ value to compare Models 2 and 3 yielded a *p*-value of 0.822, showing no difference between Model 2 and Model 3 for scalar invariance. The goodness-of-fit indices for factor variance equivalence were as follows: Q = 1.897, TLI = 0.910, CFI = 0.911, and RMSEA = 0.062. The results of using the χ^2^ value to compare Models 3 and 4 (⊿χ^2^ [df =, N = 239] =8.26), *p* < 0.05, showed that the models differed. Comparing the differences between TLI, CFI, and RMSEA in Models 3 and 4, showed that the differences were insignificant (⊿TLI = 0.001, ⊿CFI = 0.002, and ⊿RMSEA = 0.001).

As a result of the above, we confirmed the homogeneity verification of the parent–coach interaction measurement tool and showed that it can be applied equally to parents and coaches. We also confirmed configural invariance, measurement invariance, scalar invariance, and factor variance equivalence (Table 4).

#### 3.2.2. Latent Means Analysis

To perform a potential mean analysis, all measurement models’ configural invariance, measurement invariance, and scalar invariance must be established [24]. In this study, we zero-fixed the parents’ group and compared the coaches’ group with the latent mean. In the latent mean, the value (+, −) is determined by which group is fixed as the latent mean, and therefore, even the effect size value (−) cannot be interpreted as a small size. Among the potential mean comparison analyses of parent–coach interactions, no statistically significant differences were found in all subfactors (communication, talent, and support) of the parent–coach interaction measurement tool for middle and high school golfers (*p* > 0.05) (Table 5).

## 4. Discussion

The purpose of this study was to develop an interaction measurement tool for parents and coaches of middle and high school golfers, as well as to confirm its validity. In addition, two research questions to clarify the purpose of the study were formed. Based on the results of a series of research procedures and analyses, the following discussion is presented.

Based on our group results [17], after conceptualizing the contents of the parent–coach interaction tool, 40 items were produced through a pilot test, and after excluding 15 that did not meet the criteria, 25 items were investigated. Exploratory factor analysis showed an interaction factor structure of parents and coaches extracted from 20 items of three factors: communication, talent, and support. Communication and support were also commonly expressed in the interaction between athletes and parents [15], and parents and coaches recognized that mutual respect, trust, consensus, and overall support were important.

This study found that it was necessary to include understanding, sharing, dialogue, and trust-related to communication, which is attributed to the need for a process of consensus and mutual respect through dialogue based on basic courtesy. Poczwardowski et al. [25] also emphasized the importance of communication by reporting that smooth communication through dialogue with interest can have a positive impact on individuals and team members. The ultimate goal of parents and coaches is to improve athletes’ performance, consistent with the study by Stephan [20], who mentioned the need for communication between athletes, parents, and coaches. Communication is very important in parent and coach interactions, and it can provide sufficient meaning to the discovery of communication factors in the measurement tool development stage.

Qualification-related items included the role of coaches, education issues, and the content of guidance methods. In detail, it consisted of the coaches’ ability, personality, and coaching methods that were strongly required from the coach from the parents’ point of view, and the coaches also judged that they needed the right education and qualifications to coach the players.

A recent viewpoint presents that coaches’ qualities are not limited to professionalism and ability, but their personality as well. This is because the morality, honesty, and responsibility of coaches can also affect learners’ educational aspects. In the field of physical education, it is also important to create an environment for athletes to cultivate desirable qualities through proper education, rather than limiting their coach’s abilities and role to improving their athletic performance. External parties should keep in mind that coaches’ qualities and roles can affect student-athletes in their growing years, as their values are still not established. The fact that coaches’ personal abilities, views, and philosophy were extracted as talent factors in parent and coach interactions suggests that they can be an important variable in the educational field.

The support factors in the interaction between parents and coaches consisted of items related to interpersonal relationships, career paths, college entrance, and psychological support [26]. Student-athletes are not yet adults, and they are dependent on their parents and coaches for continuous support. Athletes need their parents to provide a proper environment and focus on economic support and exercise and want their coaches to provide psychological stability and efficient training.

It is noteworthy that in the field of education, information support is important for careers and college entrances. Parents not only support their children to concentrate on sports, but actively collect opinions from coaches in terms of careers and entering college. It is estimated that these results are related to the factors of communication described earlier. This is because smooth communication through understanding, dialogue, and trust between parents and coaches is needed to provide positive information on career paths and entrances for student athletes. Overall support for student athletes is an indispensable area, and parents and coaches also recognize the need and importance of such support. Therefore, the support factor extracted through exploratory factor analysis can be interpreted as a meaningful result. We conducted a confirmatory factor analysis with 20 items of the three factors extracted from the exploratory factor analysis. Model 1 with the single factor of 20 items was found to be unsuitable (TLI: 0.842, CFI: 0.859), but Models 2 and 3 were found to be suitable. Therefore, the three primary factors are considered to be highly descriptive by one secondary factor and are interpreted as confirming the validity of the theoretical structure.

In the process of securing the external validity of the developed measurement tool, it was confirmed that the tool could be applied equally to parents and coaches. These results identify the structure of interaction measurement tools for coaches and parents of middle and high school golfers, which can be applied to the two groups in common. Furthermore, it can be used as a resource material for research that explores parent–coach interactions in various sports, including golf.

As construct equivalence was identified, potential average comparisons considering measurement errors were conducted as part of securing the tool’s external validity. We checked for differences in the communication, talent, and support factors, and found no significant differences in all factors (*p* > 0.05). However, by applying Cohen’s effect size (d) [27], we can see that there exists a difference between parents and coaches, although there is no significant difference in support factors, and a small size (d) value can be identified.

In sum, the measurement of the interaction between parents and coaches of middle and high school golfers includes communication, personal ability, and personality of coaches, including courtesy, trust, and faith. In addition to the qualities of a coach that imply efficient coaching methods, information on entering college, and psychological support, as well as the structural concept of interaction between parents and coaches, are sufficiently meaningful. Through the feasibility check process of the developed measurement tool, the validity and construct equivalence proof of the theoretical structure of the interaction measurement tool suggest that it can be used not only for golf but for other sports as well. Since this study developed measurement tools on parents and coaches of middle and high school student golfers, the interaction can be more clearly identified by considering the specificity of the sport and age. In addition, the relationship between parents and coaches is absolutely required in the overall life and training of athletes, so the use of parents and coaches’ interaction measuring tools will be applicable to a variety of sports, not limited to golf and age. Because the measurement tool contains what parents and coaches think of interactions, it will be able to effectively apply “information about interactions” to sport fields through brief and clear questions.

### Strengh and Limitations

This study has several limitations and suggestions for future research. First, the approach to interaction is important. As studies on the interaction among athletes, parents, and coaches continue, research is required to integrate the interaction of athletes and parents, of athletes and coaches, and of parents and coaches. This approach can lay the foundation for establishing a theoretical framework for athlete, parent, and coach triangular interactions, and expect meaningful results in terms of practical utilization. Second, the parent and coach measurement tool developed in this study demonstrated that it is equally applicable for both, parents and coaches. Therefore, in studies involving parent–coach interactions, hypothetical theoretical models should be established, and causal relationships should be identified to provide effective information in this field. Third, this approach provides fundamental data that can be applied not only to golf but also to a variety of sports events, suggesting a direction for future research. In addition, this result allows convenient measurement of the interaction between parents and coaches. Therefore, we hope that these studies can be actively used in the future to review causal relationships with various related variables.

## 5. Conclusions

This study aimed to develop a measurement tool for the interaction between parents and coaches of middle and high school golfers to provide information that can be applied in the practical field. Based on the results, the following conclusions were drawn. 

First, we developed preliminary items, removed unsuitable items, and conducted a pilot test through the conceptualization of parent–coach interaction, and produced 40 final items. After eliminating 20 items through factor analysis, reliability analysis, and exploratory factor analysis, the final three factors were extracted from 20 items, and the parent–coach interaction measurement tool was found to meet the criteria (Q = 1.776, TLI = 0.904, CFI = 0.916, RMSEA = 0.052). Second, a construct equivalence analysis was conducted to verify the external validity of the parent–coach interaction measurement tool and confirmed that the developed interaction measurement tool could be applied equally to parents and coaches. Furthermore, it was intended to provide a variety of information on parent and coach interactions by conducting a potential mean comparison.

## Figures and Tables

**Table 1:** Parent–coach interaction measuring tools preliminary items configuration and items analysis results.

	Mean	Standard Deviation	Kurtosis	Skewness
1. I try to understand the coach (parent)	3.35	1.34	−1.02	−0.28
2. I am grateful to the coach (parent)	3.26	1.10	−0.40	−0.13
3. I respect the coach (parents)	3.49	1.16	−0.37	−0.42
4. I am polite to the coach (parents)	3.26	1.06	−0.27	−0.18
5. I admire the coach (parents)	2.31	1.26	−0.72	0.566
6. I talk to the coach (parents) a lot	3.54	1.29	−0.75	−0.45
7. I have a close relationship with the coach (parents)	4.03	1.17	0.41	−1.11
8. I greet the coach (parents) with a smile	2.17	1.25	−0.40	0.80
9. I can communicate well with the coach (parents)	3.78	1.06	−0.04	−0.65
10. I consult with the coach (parents)	2.05	1.17	0.19	0.963
11. Information are shared about coaches (parents) and athletes (children)	3.48	1.04	−0.27	−0.33
12. I meet the coach often (parents)	2.46	1.24	−0.64	0.48
13. I enjoy mutual trust with the coach (parents)	3.96	1.12	0.17	−0.91
14. I trust the coach (parent)	2.79	1.24	−0.74	0.154
15. Coaches (parents) are interested in the entry of athletes (children)	1.95	1.16	0.35	1.10
16. Coaches (parents) are interested in the career path of athletes (children)	3.75	1.10	−0.20	−0.57
17. Coaches (parents) present future directions to athletes (children)	3.71	1.36	−0.66	−0.72
18. Coaches (parents) seek psychological stability for athletes (children)	3.65	1.24	−0.70	−0.50
19. Coaches (parents) care about stress management for athletes (children)	2.36	1.41	−0.97	0.56
20. Coaches (parents) strengthen the willpower of players (children)	3.50	1.25	−0.74	−0.41
21. Coaches (parents) value the interpersonal relationships of athletes (children)	3.46	1.23	−0.69	−0.31
22. Coaches (parents) value their relationships with peers	3.96	1.18	0.14	−0.99
23. Coaches (parents) believe that economic support is necessary	2.43	1.35	−0.88	0.53
24. Coaches (parents) think voluntary support is necessary	3.20	1.21	−0.74	−0.10
25. Coaches (parents) prioritize the performance of athletes (children)	2.49	1.42	−1.07	0.45
26. Coaches (parents) value the skill acquisition of athletes (children)	3.91	1.30	−0.28	−0.92
27. Coaches (parents) try to ensure that athletes (children) adapt well to training	3.59	1.18	−0.39	−0.49
28. Coach (parents) often say positive things to players (children)	3.65	1.31	−0.66	−0.61
29. The coach (parent) values the performance of the competitors (children)	2.07	1.25	−0.35	0.84
30. Coaches (parents) try to keep their children (athletes) exercising	3.05	1.37	−1.10	−0.00
31. Coaches (parents) have a sense of responsibility	3.89	1.29	−0.26	−0.91
32. Coaches (parents) are punctual	3.40	1.26	−0.73	−0.35
33. Coaches(parent) are honest	1.66	1.16	1.94	1.74
34. Coaches (parents) are interested in improving the quality of education	3.72	1.27	−0.62	−0.66
35. Coaches (parents) pursue effective teaching methods	3.93	1.21	−0.22	−0.87
36. Coaches (parents) value learning consistency	4.27	1.09	1.31	−1.44
37. Coaches (parents) are faithful to their roles	3.89	1.20	−0.09	−0.87
38. Coaches (parents) are coercive with their children (athletes) (R)	2.20	1.27	−0.37	0.81
39. Coaches (parents) have an authoritative attitude (R)	2.24	1.26	−0.53	0.68
40. The coach ignores me (R)	3.72	1.15	−0.47	−0.53

**Table 2:** Exploratory factor analysis of coach–parent interaction.

Factor	Item No.	Factor 1	Factor 2	Factor 3
Communication	2.	0.733	0.231	0.005
	4.	0.706	0.179	0.234
	11.	0.655	0.135	0.165
	9.	0.646	0.186	0.190
	1.	0.532	0.278	0.178
	13.	0.482	0.305	0.298
	6.	0.412	−0.006	0.330
Talent	31.	0.004	0.623	0.120
	33.	0.315	0.594	0.109
	32.	0.180	0.590	0.247
	40.	0.358	0.585	−0.013
	34.	0.146	0.568	0.259
	35.	0.115	0.551	0.213
	37.	0.274	0.526	0.114
Support	26.	0.045	0.048	0.773
	27.	0.234	0.167	0.675
	16.	0.134	0.379	0.569
	28.	0.293	0.365	0.532
	17.	0.257	0.252	0.518
	22.	0.342	0.269	0.412
Eigenvalue		6.528	1.302	1.196
% of Variance		32.642	6.510	5.980
% of Cumulative		32.642	39.152	45.132

Sample suitability measurements of Kaiser–Meyer–Olkin = 0.903. Sphericalness verification of Bartlett = 1684.223, df = 190, sig = 0.000.

**Table 3:** Confirmatory factor analysis goodness-of-fit index.

Model	χ^2^	P	DF	Q	TLI	CFI	RMSEA
Model 1 (single-factor)	387.324	0.000	170	2.278	0.842	0.859	0.067
Model 2 (three-factor)	296.509	0.000	167	1.776	0.904	0.916	0.052
Model 3 (hierarchical)	296.509	0.000	167	1.776	0.904	0.916	0.052

**Table 4:** Homogeneity verification goodness-of-fit index of parent–coach interaction.

Model	Chi-Square	df	Q	TLI	CFI	RMSEA
configural invariance	648.512	334	1.942	0.906	0.917	0.063
measurement invariance	687.188	351	1.958	0.904	0.911	0.064
scalar invariance	701.337	371	1.890	0.911	0.913	0.061
factor variance equivalence	709.601	374	1.897	0.910	0.911	0.062

**Table 5:** Latent mean results of parent–coach interaction.

Factor	Parent		Coaches		*p*	Effect Size (d)
	LM	M (SD)	LM	M (SD)		
Communication	0	3.46 (0.70)	0.012	3.47 (0.75)	0.901	0.024
Talent	0	3.72 (0.80)	0.049	3.76 (0.71)	0.647	0.079
Support	0	3.78 (0.73)	0.042	3.82 (0.69)	0.606	0.120
